# Comparative Medical Ethnobotany of the Senegalese Community Living in Turin (Northwestern Italy) and in Adeane (Southern Senegal)

**DOI:** 10.1155/2012/604363

**Published:** 2012-06-18

**Authors:** Rachele Ellena, Cassandra L. Quave, Andrea Pieroni

**Affiliations:** ^1^University of Gastronomic Sciences, Piazza Vittorio Emanuele 9, Pollenzo, 12060 Bra, Italy; ^2^Center for the Study of Human Health, Emory University, 550 Asbury Circle, Candler Library 107, Atlanta, GA 30322, USA

## Abstract

A medico-ethnobotanical survey was conducted among the Senegalese migrant communities of Turin (Piedmont, NW Italy) and their peers living in Adeane (Casamance, Southern Senegal), both among healers and laypeople. Through 27 in-depth interviews, 71 medicinal plant taxa were recorded and identified in Adeane and 41 in Turin, for a total of 315 different folk remedies recorded in Senegal and 62 in Turin. The large majority of the medicinal plants recorded among Senegalese migrants in Turin were also used in their country of origin. These findings demonstrate the resilience of home remedies among migrants and consequently the role they should have in shaping public health policies devoted to migrant groups in Western Countries, which seek to seriously take into account culturally sensitive approaches, that is, emic health-seeking strategies.

## 1. Introduction

In the last decade, the ethnobotany of migrant populations, especially in Western countries, has become the focus of a number of studies, which have investigated the trajectories of change of Traditional Medicines (TMs) and especially Traditional Knowledge (TK) concerning medicinal plants. Moreover, such studies have made progress in gaining a better understanding of newcomers' health-seeking strategies. These data are crucial in the implementation of culturally sensitive approaches in public health and nutritional policies in the host countries and/or to improve phyto-pharmacovigilance [1–5].

In particular, in Europe, the ethnobotanical knowledge of various migrant groups has been studied in different (mainly urban) contexts: Turkish and Russian migrants in Germany [6, 7]; Thai women in Sweden [8]; Surinamese migrants in The Netherlands [9, 10]; South-Asians [11–14] and Andeans in England [15–17]. From these previous studies, three key findings have emerged so far.

- Newcomers' TK and related domestic practices may show various degrees of resilience (i.e., the attitude to recover from the changes, which originate from the displacement).
- The resilience is highly dependent on practical circumstances (distance between the home and the host countries, corresponding to possibilities of frequent travel), but also on complex cultural exchanges ongoing between the diasporas and the autochthonous and/or other migrant populations. For example, factors such as (1) the occurrence of relevant transnational social and trade networks between the migrants and their home country, (2) the availability of traditional practitioners and/or herbs and food plant items in food shops in the host country, (3) identity-bound perceptions in relation to specific botanicals (which may be considered culturally important), (4) laws in place in the host countries allowing or tolerating the occurrence of non-autochthonous food/medicinal plants, and (5) multicultural approaches in the institutionalised public health frameworks of the host country, all play crucial roles in determining the resilience and sustainability of these TM practices in the migrants' host country.
- The aforementioned cultural negotiations that impact TK resilience are rapidly changing on both temporal and spatial scales, and even the “representation” of plants and remedies related to “traditions” is in a state of flux among generations over time.

In Italy, no ethnobotanical study has addressed these specific issues thus far, despite the fact that the country has faced tremendous changes in its social structure over the last two decades. In fact, these changes are due in large part to the arrival of a significant number of young and middle-aged migrants from Africa and especially Eastern Europe (most notably, Romanians and Albanians). Nowadays, it is estimated that five million migrants live in Italy, with an increase of three million in the last ten years [18]. The large majority of migrants live in the Central-Northern regions of the country; one-fifth of which are Romanians, followed by Albanians and Moroccans. The Senegalese are quantitatively the 17th largest migrant community in Italy, but they represent the biggest “black” African community in the country, encompassing approximately 73,000 members. Moreover, this community is also historically one of the most important migrant groups in Italy, as it formed a significant presence already in the 1980's [18].

Recent sociological studies have pointed out the existence of a Senegalese transmigrant movement made of people who are regular “comers and goers” between Africa and Europe and that their perception of a successful return is still associated—in contrast with other African communities—with permanent return to their homeland. This final aim is, however, generally compromised with aspirations of economic advancement and family obligations [19, 20]. Most of the earnings of Senegalese migrants are used for investment in housing in their home country, significantly altering the landscape of local cities [21].

Despite the fact that a study has well demonstrated the link between depression and rapid changes in the social organisation among Senegalese migrants [22], a fair public debate on culturally sensitive approaches in transcultural health policies is still lacking in Italy. This could be due to the state of political discourse in Italy, which has been highly influenced over the last years by instances of xenophobia, and which has subsequently affected several political actors and policy makers [23–27].

The aims of this study were to record uses of natural remedies (including food preparations perceived as “healthy”) among the Senegalese community of Turin (Northern Italy) and in their country of origin, to compare these two ethnobotanies and to consequently formulate considerations on how TK changed or is changing following displacement of Senegalese citizens.

## 2. Material and Methods

### 2.1. The Study Area and Fieldwork

#### 2.1.1. Turin, Italy

**Figure 1:**
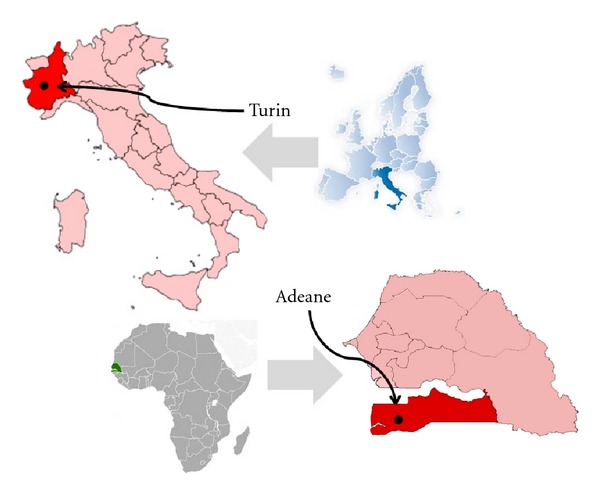
Location of the study sites.

Turin (approx. 900,000 inhabitants, Piedmont, NW Italy, Figure 1) hosts an important Senegalese migrant community counting approx. 1,200 members (2004) [28]. The most significant influx of Senegalese in Turin only began at the end of 1980s. At that time, young males migrated to Italy from various areas of the Senegalese countryside and especially from those areas which were badly affected by the great drought of the 1970s. Traditionally, families gave their fourth or fifth child away to the Islamic brotherhood of believers for instruction in the faith and to work for the order, mainly engaging in agricultural activities. With the advance of desertification, however, the practice of agriculture was increasingly difficult and, as a consequence, the order allowed young people to move abroad to work in industry and services [28].

It was therefore a progressive flow, and not a mass migration, that characterized the Senegalese emigration to Italy. As often happens, the journey for many has been fragmented at various stages due to issues such as the search of a visa or other means of entry into the country. However, in Turin, the first arrivals had no intention of staying, since their aim was to work hard for a few years and return the home country. With time, however, things have changed, resulting in more stable settlements in the Italian landscape [28].

#### 2.1.2. Adeane, Senegal

Adeane is a town of 9,000 inhabitants, located an hour's drive from Ziguinchor, the largest urban centre in the region of Casamance, Southern Senegal (Figure 1). The climate in Casamance is the most humid of the country and subtropical forests prevail in the landscape. The abundance of rain in the Casamance permits the cultivation of a wide variety of crops.

The Casamance is inhabited mainly by the Jola ethnic group (Diola, in the French transliteration), which constitute approximately 60% of the population. Those of the Wolof ethnic group, which represent the ethnic majority in Senegal, constitute only 5% in the Casamance region. The largest portion of the Casamance inhabitants identifies their religious beliefs with Islam, while 17% are Catholics. This isolation has determined a strong regional identity and thus the culture of its people as well as its environmental heritage has been well preserved for a long time. The regional economy is based in part on tourism, especially along the coast and on the sale of crops like rice, peanuts, and millet.

#### 2.1.3. The Fieldwork

Fieldwork was conducted over a period of one month (November 2010) in Turin and over a period of a second month (December 2010) in Adeane (Casamance, Southern Senegal). Turin was selected as a field site because it is the home of a vibrant Senegalese community, while the area of Casamance in Senegal was chosen because it is considered the most biological and cultural diverse region of the country, as well as the most conservative in terms of folk practices.

Participants in Turin were selected using snowball techniques among the first generation of Senegalese migrants (*n* = 8, all males), while in Casamance the same technique was used to select “laypeople” (*n* = 15, 7 females and 8 males). Additionally, in Adeane 4 healers (3 males and 1 female) were also interviewed. Prior Informed Consent (PIC) was obtained verbally before commencing each interview. Ethical guidelines followed the International Society of Ethnobiology Code of Ethics [29].

Questions concerning the use of medicinal and/or food plants were asked via a previous free listing of pathologies and related use of “home remedies.” For each named item, the field researcher (RE) asked for exact details of how the home medicine/food was prepared and its folk medical/food use. Interviews were conducted in Italian in Turin and in French in Casamance.

In Casamance, the named plant items were collected, when available, photographed, dried, identified by a local plant taxonomist (Professor Amadou Tidiane, Department of Agricultural Studies, University of Ziguinchor, Senegal) and via the West African plants photo database [30], and deposited at the Herbarium of the University of Gastronomic Sciences, Pollenzo, Italy. The nomenclature follows IPNI [31], with family assignments following the current Angiosperm Phylogeny Group III recommendations [32, 33].

### 2.2. Data Analysis

 The ethnobotanical data collected from Turin and Adeane were compared with each other. Moreover, the ethnobotanical data were compared with the preexisting literature on Senegalese TM and the traditional pharmacopoeia of Senegal [34–36].

## 3. Results and Discussion

### 3.1. The Medical Ethnobotany of the Senegalese Migrants in Turin

Table 1 reports all medicinal plants quoted by the Senegalese migrants in Turin. In total, 47 folk taxa were recorded as medicinally used in Turin; 41 of these have been botanically identified. Of these remedies, only a few (eight) could be considered food medicines, thus contradicting what previous studies among migrants medical ethnobotanies have found [6, 13, 17]. This may be due to the fact that regular provision of African vegetables and other fresh food ingredients is scarce in Turin, where generally only dried spices and medicinal plants are imported. Another explanation may be that the Senegalese migrant community in Turin is mainly represented by males, who—in contrast to women—are not holders of culinary knowledge and therefore they do not generally have experience in managing healthcare via the diet within the domestic domain.

All remedies quoted in Turin are generally bought in small ethnic food shops and mini-supermarkets located in city centre and managed by African and/or Chinese migrant entrepreneurs. A few of the most quoted taxa (*Acacia, Adansonia, Guiera, Hibiscus*) are well-known African medicinal plants, which are however lacking in the Western TM pharmacopoeia.

### 3.2. The Medical Ethnobotany of Adeane in Senegal

Table 2 reports all medicinal plants quoted in Adeane. In total, 71 species, representing 31 botanical families, were recorded as components to TMs in Adeane. However, although the large majority of recorded medicinal taxa were found in the reviews of the Senegalese TM [34–36], only a minority (<40%) of the actual medicinal plant uses are reported in the considered literature. This confirms the highly dynamic character of the home medicines in rural Africa and highlights the urgent need for inventorying folk plant uses beyond those that are cited in the “standardized” TM reviews.

**Figure 2:**
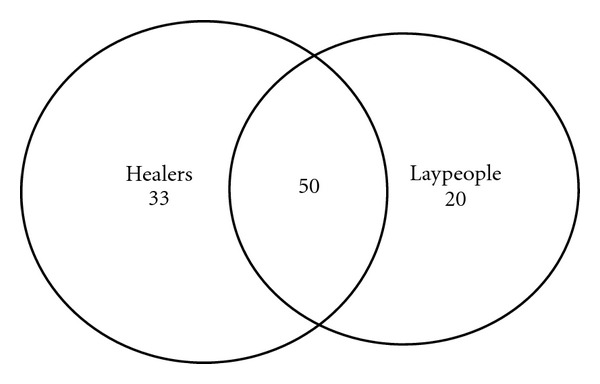
Overlap between the folk medicinal taxa quoted by healers and laypeople in Adeane.

**Figure 3:**
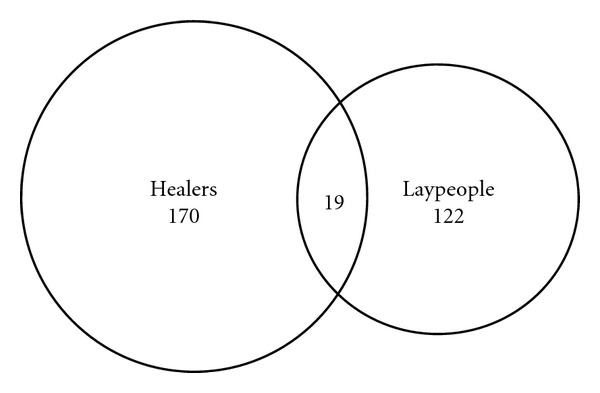
Overlap between the medicinal plant reports quoted by healers and laypeople in Adeane (a single medicinal plant report is defined as “*a given taxon x*, *prepared as y*, *used for z*”).

Documentation and evaluation of these home remedies are very important, since they represent a means of primary healthcare for most.  Figure 2 illustrates the overlaps between the plants quoted in Casamance by healers and laypeople. Laypeople's knowledge of medical plants is quite remarkable and confirms that the actual practice of household phytotherapy in Africa is much broader of what we sometimes label as “Traditional Medicine,” which is generally restricted to the knowledge, practices, and beliefs of healers. Moreover, despite living in the same village, while healers and laypeople use in large part the same medicinal plants (Figure 2), the actual plant reports (plant-based preparations used for a given health problem) are highly divergent (Figure 3). These findings confirm a remarkable “internal” variability of the African medical ethnobotanies, as a recent study in rural Mozambique also pointed out [37].

### 3.3. Comparison between the Senegalese Medical Ethnobotanies of Turin and Adeane

**Figure 4:**
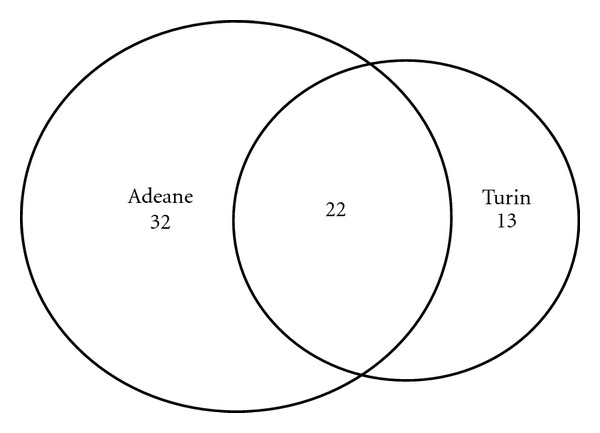
Overlap between the botanical genera quoted as medicinally used in Turin and Adeane (by laypeople).

A comparison between the laypeople's medical ethnobotany in Turin and Adeane demonstrates that Senegalese in Senegal use more plants than Senegalese in Turin (Figure 4). This may be due to an objective difficulty to acquire all African plants used in country of origin in the new cultural environment in Italy and also to an adaptation process. Migrants moved in fact from their original rural areas in Senegal (where the use of herbal TMs is widespread) to urban environments in Europe, where practices of use of medicinal plants are only available within the context of Western modern herbalism and phytotherapy: Senegalese TM practitioners seem in fact not to be present in Turin. Moreover, migrants from Senegal in Turin also generally rely on Western pharmaceuticals.

However, the large majority of the medicinal botanical genera recorded in Turin are also used in the country of origin, thus confirming some resilience of original practices following displacement into another landscape. The fact that a few other genera (twelve) have been quoted instead by migrants in Turin, but not in Adeane, could possibly be explained in two ways.

- Senegalese migrants living in Turin did not all come from the southern part of Senegal. For instance, a few of them may have brought plant uses to Turin that are unknown in the folk medicine of Southern Senegal.
- A few genera recorded quoted in Turin (i.e., *Hibiscus*, *Zingiber*) may represent the result of cross-cultural exchanges of TMs with other migrant populations in Turin, especially with the North African migrants, who also share the same religion, and with members of the Chinese migrant community who own ethnic food markets in Turin.

Out of this comparative study, a few plant families have emerged as being integral to the TM practices of the Senegalese study participants both in Turin and Adeane. In particular, a great variety of Fabaceae species were quoted as having medicinal applications in Turin (7 species) and Adeane (15 species). The second and third most represented botanical families amongst the Turin participants were Combretaceae and Malvaceae, with 5 and 3 species quoted, respectfully. In Adeane, however, Malvaceae was the second most quoted family (5 species), followed by Apocynaceae and Solanaceae (4 species each), and then Combretaceae, Myrtaceae, Euphorbiaceae, and Meliaceae, represented by 3 species each.

Interestingly, despite the presence of a thriving Senegalese community in the north Italian landscape for more than 30 years, relatively few Italian medicinal plants appear to have been incorporated into the TM practices of this group. Take, for example, the notable lack of incorporation of several European mints (Lamiaceae) in the TM practices of the Senegalese in Turin. Various Lamiaceae species, such as mint, basil, peppermint, rosemary, thyme, horehound, and oregano, grow in the wild and/or are cultivated in the Italian countryside and the use of such species for medicinal purposes dates back to more than 2,000 years ago in this region, as evidenced by their presence in the ancient textbooks of the Mediterranean *Materia Medica *[38]. Moreover, the important use of Lamiaceae species as medicinal plants is crucial also in the medico-ethnobotanical literature of Piedmont ([39] and references therein). The conspicuous absence of Lamiaceae uses in the Senegalese migrant community is maybe reflective of their isolation from the Italian environmental and medical landscape, which may have been further enhanced by the characteristic male composition of the Senegalese community in Italy.

## 4. Conclusion

Our study illustrates that the herbal medicines used by the Senegalese in Turin are very different from those of the Italian herbal landscape and that the migrant population in Turin is instead reliant on the undependable trade and movement of plant materials from their homeland to ethnic markets in the city. This shows maybe a scarce integration of this African community into the host society.

Moreover, the access and availability of important original medicines, especially medicinal foods, are greatly diminished in Turin, creating a significant disruption in their TM system. This may also have been influenced by the general lack of female Senegalese migrants, who would typically be the ones in charge of TM and “health” foods in the domestic setting.

The issues relevant to primary TM practice in migrant communities are often compounded by a lack of specific health policies, which are able to address migrant needs. This problem is, of course, not isolated to the case of migrants in Italy, but is also relevant to many other Western countries, where the healthcare needs of burgeoning migrant populations are often conspicuously absent in health policy and legislation.

By having a better understanding of both the migrant folk pharmacopoeia and the state of TK transmission with regards to health, more culturally sensitive health policies could be developed. In particular, the increasing occurrence of newcomers in Italy should foster more pluralistic approaches in the management of CAMs by the regional authorities, as well as consequently addressing measures aimed to improve the information on potentialities and risks of “home-made” herbal remedies.

## Figures and Tables

**Table 1:** Medicinal plant remedies used by Senegalese migrants in Turin.

Botanical taxon, family, and voucher specimen code	Local name(s)	Part(s) used	Preparation and administration	Folk medical use (used against/to regulate)	Qs
*Adansonia digitata *L. (Bombacaceae)	Baobob	Seed	Eaten	Diarrhoea	+

		Seed	Grind the seeds and put the powder on the lips	Burning lips	
			Grind the seeds and put the powder on the painful tooth	Toothache	
*Acacia nilotica* (L.) Willd. ex Delile (Fabaceae) UNISGSEN15	Mbano (m) Nep nep (w)	Root	Make a decoction and drink just a little bit	Indigestion	+++
			Externally applied	Wounds	
		Bark	Drink the beverage together with *Tamarindus indica* fruit pulp and *Hibiscus sabdariffa* flowers	Fatigue	

			Grind it, add salt, and put it on the haematoma	Haematoma	+++
*Acacia tortilis *(Forssk.) Hayne (Fabaceae) UNISGSEN05	Senjen (w)	Root	Drink the decoction	Eye inflammations Bellyache	
			Drink the decoction while eating some sugar, repeating the procedure three times a day	Worms	
			Macerated in water for two days; the macerate drunk	Sexual impotence Stomachache Kidney troubles	

*Adansonia digitata* L. (Malvaceae) UNISGSEN20	Buy (w) Baobab (f)	Fruit	Drink the beverage made using the pulp around the seeds	Diarrhoea	+++
			Same as above, adding* Hibiscus sabdariffa *flower	Diarrhoea	

*Allium cepa* L. (Amaryllidaceae)	Cibolle	Bulb	Eaten	Sexual impotence Cold	+

*Allium sativum* L. (Amaryllidaceae)	Ail (f) Ladji (w)	Bulb	Put a piece of garlic on the right wrist if the sore tooth is in the upper jaw and vice versa for the lower jaw (chanting Koran's verses helps the pain to disappear)	Toothache Sexual impotence	+++
			Eaten	Intestinal worms Lowering the blood pressure Cold and cold prevention	

*Anacardium occidentale *L. (Anacardiaceae)	Anacardo	Seed	Drunk	Antibiotic	+

*Annona senegalensis *Pers. (Annonaceae)	Suncun	Leaf	Put (powdered) on the fire	Magic remedy (supposed to counteract bad spirits)	+

*Arachis hypogaea* L. (Fabaceae) UNISGSEN16	Arachide (f) Gerte (w)	Seed	Eat the seeds	Stomachache	+

*Balanites aegyptiaca * (L.) Delile (Zygophyllaceae)	Dattier du désert (f) Soumpu Gurp Petit cola	Leaf	Grind the leaves and drink the infusion prepared with the powdered leaves	Sore throat	+++
		Fruit	Chew the fruit without ingesting it	Stomachache Digestive Fatigue Nausea	

*Calotropis procera* (Aiton) W.T. Aiton (Apocynaceae) UNISGSEN55	Kipampaan (p) Poftan (m) Pomme de Sodome (f)	Root	Cut the roots into small pieces, put it into a cotton handkerchief, squeeze, it and inhale the aroma	Sinusitis	+
		Stems juice	Externally applied	Wounds	

		Leaf	Drink the infusion prepared with ground leaves	Constipation	+++
*Cassia italica *(Mill) Sprengel (Fabaceae)	Layduur (w)		Drink the infusion prepared with ground leaves	Intoxication	
		Root	Leave the root soaking all night and drink the water in the morning, before breakfast	Intestinal worms	

*Cassia occidentalis* L. (Fabaceae) UNISGSEN11	Adiana (w) Bantaare (p) Bentamarè (s) Kassala (m) Mbanta xobi (w)	Leaf	Put leaves around the head	Headache	+
			Baths	Fatigue	

*Cassia tora* L. (Fabaceae) UNISGSEN76	Cassepuante (f) Ndur (w)	Leaf	Use it as a mouthwash	Mouth infections	+
*Ceratotheca sesamoides * Endl. (Pedaliaceae)	Jorokh lane (w)	Leaf	Put the leaf into water at room temperature and after it releases oil, apply the oil to the body	High fever	+
			Dried leaf is soaked in water; the water is drunk	Bellyache	

*Citrus limon *(L.) Osbeck. (Rutaceae)	Citron (f) Limon (w)	Fruit	Drink the juice	To lose weight Digestive Strengthening Malaria (drunk in the coffee)	++

*Combretum aculeatum * Vent. (Combretaceae)	Sawat (w)	Leaf	Put ground leaves and sugar into water and instil the solution in the eyes	White spot in the eye on the pupil	+

*Combretum glutinosum * Perr. ex DC. (Combretaceae)	Chigommier (f) Rat (w)	Leaf	Drink the decoction	Bronchitis/cough Sexual impotence	+

		Leaf	Drink the decoction (sometimes adding cloves)	Cold Flue Lung infections Sore throat Antihypertensive	+++
*Combretum micranthum * G. Don (Combretaceae) UNISGSEN10	Quinkeliba (f) Sekhaw (w)	Flower	Drink the decoction with milk every morning	Enhancing the “well-being”	
			Make a decoction with *Xylopia aethiopica *seeds and cloves (*Eugenia caryophillata *flower buds)	Vision problems	

*Dioscorea *spp. (Dioscoreaceae) UNISGSEN83	Igname (f) Yam (w)	Root	Eat the root	To gain weight	+

*Elaeis guineensis *Jacq. (Arecaceae)	Palmier à huile (f) Tiir (w)	Fruit→ Oil	Add oil and coffee to *Vitellaria paradoxa* butter and dab on the body	To get rid of the “dead” blood when feeling weak	+
			Dab on the body	Boils	

*Eucalyptus globulus *Labill. (Myrtaceae) UNISGSEN88	Eucaliptus (f) Khotta bu tel (w)	Leaf	Put it around the head while listening to the reading of the Koran	Headache	+

*Eugenia caryophyllata* Thunb (Myrtaceae) UNISGSEN38	Girofle (f) Xorompole (w)	Flower bud	Use the infusion prepared with *Xylopia * *aethiopica* seeds and* Combretum micranthum* flower	Vision problems	+

*Euphorbia balsamifera * Ait. (Euphorbiaceae)	Salan Salan mbechi	Branch	Cut the branch and put the latex on the wound	Wounds	++

		Root	Drink the decoction	Bellyache	
*Ficus iteophylla* Miq. (Moraceae)	Xassum loro (w)	Leaf	Drink the decoction	Digestive asthma	+++
		Leaf	Dab on the affected part	Backache skin allergies	

*Grewia bicolor* Juss. (Malvaceae)	Kel (w)	Leaf	Drink the decoction	Fatigue Bronchitis/cough Digestive	++

*Guiera senegalensis * J.F. Gmel. (Combretaceae) UNISGSEN18	Ngueer (w) Mamakumkoyo (m) Mamankuiò (s)	Root	Drink the decoction	Cold	+++
		Leaf	Drink the decoction	Bronchitis/cough kidney troubles Fatigue stomachache	

			Put the infusion into the eyes	Itchy eyes	+++
		Flower (red)	Drink the decoction	Bellyaches Menstrual pains Preventing ageing Fatigue Fever Improving the blood circulation	
*Hibiscus sabdariffa* L. (Malvaceae) UNISGSEN12	Bissap (w) Karkadè (f)		Put the infusion into the eyes	Eye problems	
			Drink the beverage together with *Tamarindus indica* fruit pulp and *Acacia nilotica *bark	Fatigue	
		Flower (white)	As a food medicine—as a main ingredient of a dish prepared with boiled meat of fish, cooked with tamarind and chilies (*lakk bissap*)	Fatigue	

*Maerua crassifolia* Forssk (Capparaceae)	Sothiou (w) Sothiou suukar	Branch	Chew the branch	Halitosis	+

*Mangifera indica* L. (Anacardiaceae) UNISGSEN37	Manguier (f) Màngo joolaa (w)	Leaf	Drink the decoction	Tetanus	++

*Manihot esculenta * Crantz (Euphorbiaceae) UNISGSEN07	Gnambi (w) Manioc (f) Mañok (m)	Root	Eat the root	To gain weight	+

*Moringa oleifera *Lam. (Moringaceae)	Nebedai	Leaf	Eaten in sauces, generally accompanied with meat and couscous	Diabetes	+

*Musa paradisiaca *L. (Musaceae)	Bananier (f)	Leaf	Topical application of the leaf infusion	Burns	+

*Panicum miliaceum *L. (Poaceae)	Mil (f)	Fruit	Eat millet (couscous)	To gain weight Fatigue	+

*Parinari macrophylla *Sabine (Chrysobalanaceae)	New (w) Tamba	Leaf	Drink the infusion made from 7 leaves	High blood pressure	+
		Leaf	Drink the infusion	Stomachache	

*Piper nigrum * L. (Piperaceae)	Mex pobare (w) Poivre noir (f)	Fruit	Eat the dried berry	Runny nose	+

*Tamarindus indica * L. (Fabaceae)	Tamarin (f) Daqaar (w)	Fruit pulp	Eaten, or drunk, in a beverage made adding the bark of *Acacia nilotica *and the flower of* Hibiscus sabdariffa *	Fatigue	+

*Terminalia catappa * L. (Combretaceae)	Badamier (f) Toubab (w) Xopp kerte	Not specified	Not specified	Antibiotic, antifungal	+

			Add salt to the butter and dab on the back	Backache	+++
*Vitellaria paradoxa * C.F. Gaertn. (Sapotaceae)	Karitè (f)	Seed→butter	Add coffee,* Elaeis guineensis'* oil and dab on the body	To get rid of the “dead” blood when feeling weak	
			Dab the butter on the body	Massage on the child's body, to make the child stronger. Bone strengthening	
			Dab the butter on hair	Hair loss	

			Drink the decoction, also a spice in the coffee	Sexual impotence	++
*Xylopia aethiopica * A. Rich. (Annonaceae) UNISGSEN70	Diar (w) Jar	Seed	Instill the infusion of seeds into the eyes	Eye problems, conjunctivitis	
			Instill the infusion of seeds into the ear	Otitis	
			Pour the infusion made from the seeds, *Eugenia caryophyllata *flower buds and* Combretum micranthum* flower	Vision problems	

*Zingiber officinale *Roscoe (Zingiberaceae) UNISGSEN09	Djindjer (w) Djinjeroo (m) Gingembre (f)	Fresh rhizome	Eaten, or juice drunk, or decoction	Sexual impotence Blood circulation	+++

		Leaf	Drink the decoction	Worms	
Not identified	Berbef (w)		Use the rough leaf like a sponge under the shower	Pruritus	+
		Fruit	Make a pasta and dab on the skin	Itchiness	
Not identified	Bonye			Antibiotic	+

Not identified	Khambata (w)	Leaf	Drink the infusion	Headache	+

Not identified	Ndiadame	Fruit	Cook it slowly and eat it; it is really bitter	Intestinal worms	+

Not identified	Sangol (w)	Root	Put it in water for up to two minutes and drink it (very bitter)	Intestinal worms	+
		Leaf	Soak in water and externally apply on the skin	Itchiness	

Not identified	Watenobout (w)	Branch	Put the latex that comes out of the broken branch on the wound	Wounds	+

(f): French; (m): Mandingo; (p): Pulaar; (w): Wolof; Qs: quotations: + quoted by 1 or 2 informants only; ++ quoted by 3, 4, or 5 informants; +++ quoted by 5 informants or more.

**Table 2:** Plants used as medicines in Adeane, Casamance, Southern Senegal.

Botanical taxon, family, and voucher specimen code	Local name(s)	Part(s) used	Preparation and administration	Folk medical use (used against/to regulate)	Healers	Laypeople	Qs
*Acacia albida* Delile (Fabaceae) UNISGSEN019	Kade (f) Kadd (w) Baransango	Root Bark	Instill the infusion in the eye	Vision problems	+		3

*Acacia nilotica * (L.) Willd. ex Delile (Fabaceae) UNISGSEN15	Acacia mlotique (f) Nep nep (w) Mbano (m)	Root	Apply the infusion externally	Herpes		+	2
				Toothache		+	

*Acacia seyal* Delile (Fabaceae)	Suuro	Leaf	Topical application of liquid resulting from pressed leaves	Toothache	+		1

			Soak in water for some time and drink	Tapeworm	+		11
				Stomachache	+		
				Bellyache (abdominal pains)	+	+	
			Drink the cold infusion	Rheumatism	+		
			Apply the infusion on hair	Strengthening the hair	+		
			Boiled in water and the vapour is inhaled	Cold		+	
			Cold infusion is drunk	Blood pressure		+	
*Acacia tortilis* (Forssk.) Hayne (Fabaceae) UNISGSEN05	Senjen (w)	Root	Eat it with rice	Sterility		+	
			Make aerosol with the infusion?	Lung cancer		+	
			Drink the infusion	Bloodstream		+	
			Eat it with rice	Ulcer		+	
			Eat it with rice	Gastritis		+	
			Eat it with rice	Kidney problems		+	
			Drink the infusion	Menstrual pain		+	
				Malaria		+	
			Topical application of the infusion	Herpes		+	
			Topical application of the infusion	Boil		+	
			Drink the infusion	Fatigue	+	+	
			Drink the infusion	Tuberculosis	+	+	

		Leaf	Powdered leaf applied to burn and bandaged together with the oil of *Arachis hypogaea *	Burns	+		12
			Drink the seed juice together with the flowers of *Hibiscus sabdariffa *	Fatigue	+	+	
*Adansonia digitata * L. (Malvaceae) UNISGSEN20	Baobab (f) Guy (w)	Seeds	Eat the seeds	Diarrhoea	+	+	
			Eat the seeds together with *Citrus limon* juice	Lack of appetite	+		
			Use it with *Acacia albida, Guiera senegalensis, Parkia biglobosa, Annona senegalensis, Soora *(nonidentified plant), and* Ficus sycomorus *	Headache, sore throat, cold taken as result of wind		+	

*Allium cepa* L. (Amaryllidaceae)	Oignon (f)	Bulb	Infusion with leaves of *Citrus limon *	Sore throat	+		1

			Drink the infusion in the morning	Blood pressure	+		8
*Allium sativum* L. (Amaryllidaceae)	Ail (f) Ladji (w)	Bulb	Eat it raw in the morning	Intestinal worms	+		
			Eat it while marabout recites verses of the Koran	Depression		+	

*Aloe vera* (L.) Burm. f. (Xanthorrhoeaceae)		Leaf gel	Use it with *Vitellaria paradoxa *	Tuberculosis		+	2
			Use it with *Vitellaria paradoxa *	Hair loss	+		

			Drink the infusion	Toothache		+	3
*Anacardium occidentale* L. (Anacardiaceae)	Pomme-cajou (f) Darkasa (w) Bara diamboo (m)	Bark		Rheumatism	+		
			Put it in cold water for a while and drink it	Toothache		+	
				Blood pressure		+	

			Drink the infusion	Diabetes		+	3
			Put the powder on the wound	Wounds		+	
			Drink the infusion	Rheumatism		+	
			Drink the infusion	Bloodstream		+	
*Annona senegalensis* Pers. (Annonaceae) UNISGSEN36	Sunkun (m)	Leaf	Drink the infusion with *Musa paradisiaca* leaves and *Combretum micranthum *	Blood pressure		+	
			Use it with *Piliostigma reticulatum *	Headache		+	
			Use it with *Piliostigma reticulatum *	Tuberculosis		+	
			Use it with *Acacia albida, Guiera senegalensis, Parkia biglobosa, Adansonia digitata, Soora *(nonidentified plant), and* Ficus sycomorus *	Headache, Sore throat, Cold taken as result of wind		+	
		Leaf	Use it with *Acacia albida* and *Parkia biglobosa *	Depression		+	

		Fruit	Use it with oil of *Elaeis guineensis'* oil	Hair	+		3
*Arachis hypogaea* L. (Fabaceae) UNISGSEN16	Arachides (f) Gerte (w) Jamba katalig (m)	Leaf	Inhale the infusion prepared together with the leaves of *Mangifera indica *	General health		+	
		Seed→oil	Mix peanut oil together with the powdered leaves of *Adansonia digitata *and apply to burns before bandaging	Burns	+		
		Fruit	Eat the fruit fresh, not toasted	Cold	+		

			Wrap the leaf around the head	Headache	+		3
*Azadirachta indica* A. Juss. (Meliaceae) UNISGSEN63	Cassia, Neem (f) Niim, Ni va (w) Bantare (m)	Leaf	Infuse with hot water and inhale the steam	Fatigue	+		
			Topical application of the infusion	Skin problems	+		
			Use it with *Carica papaya *and *Citrus limon *	Cold	+		

			Make an infusion with shade-dried leaves	Diabetes	+	+	3
*Bambusa vulgaris* Schrad. ex J.C. Wendl. (Poaceae) UNISGSEN54	Bambou (f) Lonk (w)	Leaf	Drink the infusion with *Combretum micranthum* leaves	High blood pressure		+	
			Make an infusion, drink some of it, and make aerosol with the resting water	Bloodstream		+	
			Drink the infusion	Obesity		+	

*Beta vulgaris* L. (Chenopodiaceae)	Betterave (f) Beteraaw (m)	Root	Eat it	Bloodstream	+		1
			Eat it	Anemia	+		

*Borassus flabellifer* L. (Arecaceae) UNISGSEN89	Ronier (f) Kòoni (w)	Fruit	Burnt and pressed, pour resulting liquid into the ear	Earache		+	1

				Malaria	+		6
				Fatigue	+		
		Leaf	Warm the leaf up and wrap it around the neck	Sore neck	+		
			Topical application of juice from leaf on tooth	Toothache	+		
*Calotropis procera* (Aiton) W.T. Aiton (Apocynaceae) UNISGSEN55	Pomme de Sodome (f) Poftan (m) Kipampaan (p)		Wrap the leaf around the sore knee	Sore knees	+		
			Wrap the leaf around the head	Headache	+		
		Root	Use it with *Ocimum basilicum *	Rheumatism	+		
		Leaf ?	Use it with *Mànganaso *(unidentified plant) and *Jatropha curcas. *Drink it and vomit everything yellow	Yellow fever		+	
			Use it with *Jatropha curcas *and *Mànganaso *(unidentified plant)	Syphilis		+	

*Cannabis sativa* L. (Cannabaceae)	Marijuana	Seeds	Drink the infusion	Asthma	+		1

			Drink the infusion	Lack of appetite			5
			Drink a glass of water with a chili pepper in it	Intestinal worms	+		
*Capsicum annuum* L. (Solanaceae)	Kani (w)	Fruit	Mix lemon juice (*Citrus limo*) together with a chili pepper and gargle with the solution	Sore throat	+		
			Put in a small pan with *Jatropha curcas*, and drink	Hair loss		+	
			Put it in lemon juice (*Citrus limon*) and drink it	Constipation	+		

			Eat up the oil that comes out of the nut and dab it on the body three times a day with *Vitellaria paradoxa *butter	Sore back	+		9
			Mix it with oil of *Elaeis guineensis* and butter of *Vitellaria paradoxa *and then dab it on hair	Strengthening the hair	+		
			Drink a spoonful every day	Poor memory	+		
			Drink a spoonful of it	Cough		+	
			Drink a spoonful of it	Sore throat	+		
			Topical application of the oil	Eye problems	+		
*Carapa procera* DC. (Meliaceae) UNISGSEN43	Tulukuna (m)	Seed→oil	Instill oil into the ears	Earache	+		
			Drink a spoonful of it	Fever	+		
			Drink a spoonful of it	Flu	+		
			Dab it on the skin in the area over the kidneys	Kidneys problems	+		
			Drink a spoonful of it	Bellyache	+		
			Use it before breakfast	Tapeworm	+		
			Dab it on affected muscles	Sore muscles	+		
			Peel it and apply to the sore tooth	Toothache	+		8
			Use it with* Citrus limon* and *Azadirachta indica *	Cold	+		
		Root	Drink the infusion, adding salt	Syphilis (2)	+	+	

		Leaf	Drink the infusion	Anemia		+	
			Use it together with *Psidium guajava* leaves	Cystitis		+	
*Carica papaya* L. (Caricaceae) UNISGSEN21	Papayer (f) Papaayo (w)	Seeds	Dry the seeds in the sun, powder, and add to food	Intestinal worms		+	
		Fruit	Eat a soup with a ripe papaya in it together with chicken and a root of *Tinospora bakis *	Yellow fever	+		
			Boil the unripe fruit with undecorticated rice or simply eat the fruit, raw. Another remedy is to crack an egg over the unripe papaya and eat it	Yellow fever	+	+	
		Leaf	Drink the infusion together with the buds	Yellow fever		+	
			Add the following to water: dry leaves of* Musa paradisiaca, *little unripe fruits and leaves of *Citrus limon, *leaves of *Cassia occidentalis, *leaves of *Mangifera indica, *leaves of* Ziziphus mauritiana* and drink	Malaria		+	
		Fruit	Eat it together with* Parkia biglobosa* leaves	Yellow fever	+		
		Flower	Crumble flower into the water and drink	Headache	+		14
		Leaf	Wrap around the head	Headache	+		

*Cassia occidentalis * L. (Fabaceae) UNISGSEN11	Mbanta xobi or Adiana (w) Kasalaa (m) Bentamarè (s)	Leaf	Wrap around the head	Conjunctivitis	+		
		Flowe?	Drink the infusion	Menstrual pain	+		
		Leaf	Put it in water for a while and wash yourself with it	Cold (Especially for children)	+		
		Flower?	Drink the infusion	For pregnant women	+		
		Leaf	Drop juice from pressed leaves into the ear	Problems of the eardrum		+	
		Leaf	Add the following to water: dry leaves of *Musa paradisiaca, *little unripe fruits and leaves of* Citrus limon, *leaves of *Mangifera indica, *leaves of *Carica papaya*, and leaves of *Ziziphus mauritiana *	Malaria		+	

*Cassia tora* L. (Fabaceae) UNISGSEN76	Cassepuante (f) Ndur (w)			Blemishes on scalp which may extend to the whole body		+	2
			Use it with *Ficus umbellata *	Skin fungus		+	

		Fruit	Eat it	Bellyache	+		3
		Root	Put little plants' roots in water and then drink the water It is going to fizz	Fatigue	+		
*Ceiba pentandra * (L.) Gaertn. (Malvaceae)	Fromager, Kapotier (f) Bantau Bentene (w)	Bark	Use infusion made with the bark as a mouthwash	Toothache	+		
		Root?		Cancer		+	
		Bark?	Pour the infusion on the baby's head during baptism	Infant strength and protection		+	
		Root	Good for the blood because of the red colour	Blood	+	+	

		Leaf	Make an infusion together with leaves of *Allium cepa *	Sore throat	+		9
		Fruit	Drink the juice	Sore throat	+		
			Drink the juice	To lose weight	+		
			Use the juice together with *Capsicum* to gargle	Sore throat	+		
		Leaf and Fruit	Drink a beverage made with lemon leaf and fruit (*Citrus limon*)	Headache	+		
		Fruit	Put the juice on the affected part together with *Panicum miliaceum * flour	Herpes	+		
		Fruit	Drink the infusion with lemon juice (*Citrus limon*) together with two roots of the *Gossypium barbadense *	Sexual weakness		+	
*Citrus limon *(L.) Burm. (Rutaceae)	Citron (f) Limon (w)	Leaf and Fruit	Drink a beverage made with little lemons and leaves	Cold	+		
		Fruit	Drink the juice	Cold	+		
			Use it with *Carica papaya* and *Azadirachta indica *	Cold	+		
		Leaf and fruit	Add the following to water: dry leaves of *Musa paradisiaca, *little unripe fruits and leaves of* Citrus limon, *leaves of *Mangifera indica, *leaves of *Carica papaya, *leaves of *Cassia occidentalis, *leaves of *Ziziphus mauritiana *and drink	Malaria		+	
			Eaten with *Carica papaya *	Malaria Yellow fever	+		
			Use it with *Guiera senegalensis *	Asthma	+		
		Fruit	Drink the juice	Obesity	+		
			Drink the infusion together with *Gossypium barbadense *	High blood pressure	+		
			Dink the juice with honey	Liver	+		

*Cola cordifolia* (Cav.) R. Br. (Malvaceae)	Kaba/Taba	Leaf	Drink the infusion	Malnutrition		+	1

*Cola nitida* (Vent.) Schott & Endl. (Malvaceae) UNISGSEN85	Ptit cola (f) Kola (w) Goro (p)	Seed	Chew it	Stimulant	+		1

			Drink the infusion	Cough	+		2
*Combretum glutinosum * Perr. ex DC. (Combretaceae)	Chigommier (f) Rat (w)	Leaf	Drink the infusion	Bronchitis	+		
			Drink the infusion	Sore throat	+		
			Drink the infusion	Cold	+		

		Leaf	Ingested together with *Tamarindus indica *fruit pulp	Blood pressure	+	+	9
			Drink the infusion with *Musa paradisiaca's* leaf and *Annona senegalensis *	Blood pressure		+	
			Drink the infusion	Diabetes	+		
			Use it with *Tamarindus indica *	Asthma	+		
		Leaf		Obesity	+		
*Combretum micranthum* G. Don (Combretaceae) UNISGSEN10	Kinkeliba (f) Sekhaw (w)		Drink the infusion	Bloodstream	+	+	
			Drink the infusion with these leaves together with *Bambusa vulgaris* leaves	High blood pressure		+	
			Drink the infusion without sugar	Malnutrition		+	
		Flower	Drink the infusion with cloves (*Eugenia caryophillat*a) and *Xylopia aethipioca* seeds	Vision problems	+		
		Leaf	Drink the infusion	Bellyache		+	

*Dialium guineense* Willd. (Fabaceae) UNISGSEN17	Solom (w)	Bark	Make aerosol with the infusion	Asthma	+		1

*Datura inoxia* Mill. (Solanaceae) UNISGSEN91	Datura (f) Kubejaara	Leaf	Burn the wood and mix the wood ash with powdered leaf ash External Use only—highly hallucinogenic if used internally	Allergies		+	1

*Daucus carota* L. (Apiaceae)	Carotte (f)	Leaf	Drink the infusion	Breast cancer	+		1

*Dioscorea *spp. (Dioscoreaceae) UNISGSEN83	Imuam Igname (f) Yam (w)	Root	Eat it	To gain weight		+	2
		Leaf	Boil the leaves	Asthma		+	

			Put the oil on the hair	Hair	+		6
		Fruit→oil	Topical application	Toothache		+	
			Pour in the ears	Earache	+		
			Use the palm oil together with *Sooto noir* (*Ficus capensis* Thumb.?) powdered root	Liver problems		+	
*Elaeis guineensis *Jacq. (Arecaceae)	Palmier à huile (f) Tiir (w)		Attach the root of *Moringa oleifera *to the affected area and then cover the sore part with palm oil. Do not leave the root in place too long or it will cause an infection.	Rheumatism		+	
		Seeds	Wear a necklace made out of these seeds	Sore throat	+		
			Break the seed and eat the internal part	Gastritis		+	
		Root	Put them in wine and drink it	Sterility		+	
				Skin fungus	+		
		Fruit→oil	Use the palm oil together with peanuts (*Arachis hypogea*) and dab on hair.	Hair		+	
			Use it together with *Vitellaria paradoxa* butter and *Carapa procera, *dab on hair	Hair	+		
			Apply it on the cuts together with *Hessawane *	Tetanus	+		

			Drink the infusion with *Jatropha curcas *	Cough	+		1
*Erythrina senegalensis* DC. (Fabaceae) UNISGSEN34	Erythrine du Senegal (f) Dolliw fatu	Root	Drink the infusion	Syphilis		+	
			Drink the infusion	Menstrual pain		+	

*Eucalyptus globulus *Labill. (Myrtaceae) UNISGSEN88	Eucaliptus (f) Khotta bu tel (w)	Leaf	Drink the infusion	Blood pressure	+		1

			Make an infusion and use it as mouthwash	Toothache	+		3
*Eugenia caryophyllata* Thunb. (Myrtaceae) UNISGSEN38	Girofle (f) Xorompole (w)	Flower bud	Drink the infusion	Conjunctivitis	+		
			Make infusion with seeds of *Xylopia aethiopica* and *Combretum micranthum* flower and apply on eyes	Vision problems	+		

*Euphorbia balsamifera* Ait. (Euphorbiaceae)	Salan Salana Salan Mbechi	Branch	Cut a branch and put the lymph on the wound	Wounds	+		3
				Toothache	+		

*Ficus elastica *Roxb. ex Hornem. (Moraceae) UNISGSEN59	Yirif asotu	Leaf and bark	Drink the infusion	Low blood pressure		+	2
		Leaf	Drink the infusion	Cold		+	

*Ficus sycomorus *ssp. *gnaphalocarpa *(Miq.) C.C. Berg (Moraceae) UNISGSEN42	Ficus (f) Sooto (m)	Root	Drink the infusion	Fatigue	+		2
		Leaf	Powder branches and leaves together with *Jamba Saboo *leaves and drink some of it. Wash yourself with the rest of it. Do not eat fish in the meantime.	AIDS		+	

*Ficus umbellata* Vahl (Moraceae) UNISGSEN68	Ñokolokotò (m)	Leaf	Use it with *Cassia tora *	Skin fungus		+	1
		Leaf		Cough	+		4
*Gossypium barbadense* L. (Malvaceae)	Cotonnier (f) Uiten (w)	Root	Drink the infusion together with lemon juice (*Citrus limon*)	High blood pressure	+		
			Drink the infusion made with two roots together with lemon juice (*Citrus limon*)	Sexual weakness		+	

			Drink the infusion three times a day	Cough	+		13
			Chew it and put it on the sore tooth	Toothache	+		
			Dry it and use it every day	Sores	+		
*Guiera senegalensis *J.F. Gmel (Combretaceae) UNISGSEN18	Guiera du Senegal (f) Ngeer (w) Mamakumkoyo (m)	Leaf	Make an infusion together with the lemon leaves (*Citrus limon*)	Asthma	+		
			Drink the infusion with Valda pastille (industrial pastille based on menthol and eucalyptus essential oil)	Cold	+		
			Make infusion and apply to hair	Hair		+	
			Drink the infusion	Insomnia		+	
			Apply chewed leaf onto the wound or powder it and put it on	Wounds	+	+	
			Drink the infusion together with *Mànganaso*'s root (unidentified plant)	Intestinal worms		+	
			Infusion prepared with *Adansonia digitata, Acacia albida, Parkia biglobosa*, *Annona senegalensis, Soora Soora *(nonidentified plant), and *Ficus sycomorus *	Headache, sore throat, or cold taken as result of the wind		+	

		Leaf and Flower	Drink the infusion	Bloodstream	+		12
		Flower		Flu	+		
		Fruit	Make a juice together with *Adansonia digitata *seeds	Fatigue	+	+	
		Flower		Lack of appetite		+	
*Hibiscus sabdariffa * L. (Malvaceae) UNISGSEN12	Karkadè (f) Bisaab (w)		Use it with *Tamarindus indica *and *Acacia nilotica *	Fatigue	+		
		Flower	Use it with *Phaseolus vulgaris* seeds	Anemia	+		
			Use it with seeds of *Phaseolus vulgaris *	Fatigue	+		
		Fruit	Remove the seeds and squeeze the fruit juice into the eyes.	Conjunctivitis		+	
		Leaf	Make an infusion together with the young leaves of *Psidium guajava*, drink it, and eat the leaves	Diarrhoea	+	+	

*Holarrhena floribunda * T. Durand & Schinz (Apocynaceae) UNISGSEN67	Jarko (m)			Sexually transmitted diseases	+		2
			Drink the tisane	Prostate Abortion		+	

			Collect pieces of bark that face east during sunset and then drink it in 5 litres of water	Cancer	+		6
			Collect pieces of bark that face east during sunset and then drink it in 5 litres of water	Syphilis	+		
*Jatropha curcas * L. (Euphorbiaceae)	Pignon d'Inde (f) Tabanana (w) Tabanano (m)	Bark and Root	Drink the infusion together with roots of *Erythrina senegalensis *in it	Cough	+		
			Put it in a small pan with* Capsicum *and drink	Hair loss		+	
			Powder it together with *Kunjunburun* and eat it on rice	Syphilis		+	
			Use it with *Calotropis procera* and *Mànganaso *(unidentified plant)	Yellow fever		+	

			Drink a tisane made with the bark together with *Acacia tortilis. *	Intestinal worms	+	+	5
			Boil it in the water or put it into cold water for 2 hours and then drink it.	Fatigue	+		
			Make aerosol with it	Fatigue		+	
*Khaya senegalensis * (Desr.) A. Juss. (Meliaceae)	Cailsedrat (f) Xai, kay (w)	Bark	Put in water and drink it	“Makes blood”	+		
			Make cold infusion and drink	Kidney problems		+	
				Anemia		+	
			Put the bark in a bottle and drink it.	Tuberculosis		+	

*Lawsonia inermis * L. (Lythraceae) UNISGSEN69	Hennè (f) Fuden (w)	Leaf?	Soak it together with the leaves of *Psidium guajava *and then drink it. The leaves needs to be fresh, not dried.	Stomachache		+	1

*Leptadenia hastata *Vatke (Apocynaceae)	Mboom (w) Duto (m)	Leaf	Attach the leaves to the back together with hawk's bones	Kidney problems		+	1
		Root	Scrape at the bark, put it into water, and drink it	Snakebite		+	

*Lippia chevalieri *Moldenke (Verbenaceae)	Samfitò	Leaf	Drink the infusion	Boils		+	1

*Maerua crassifolia* Forssk. (Capparaceae)	Sothiou (w) Sothiou suukar	Branch	Rub the stick on the teeth	Clean teeth	+		2
			Chew the branch. Its bark tastes like sugar	Haemorrhoids		+	

		Leaf	Make an infusion, with 1.5 L water and a handful of leaves (some say to use only the ones on the floor, others to add salt to the infusion)	Tetanus	+	+	13
		Leaf	Drink infusion	Poor memory	+		
*Mangifera indica * L. (Anacardiaceae) UNISGSEN37	Manguier (f) Màngo joolaa (w)	Bark?	Hot infusion and inhale it together with peanut (*Arachis hypogaea*) leaves	General health	+		
		Bark	Make aerosol with it	Toothache	+		
		Leaf		Toothache	+	+	
		Leaf	Add to water: dry leaves of *Musa paradisiaca, *little unripe fruits and leaves of* Citrus limon, *leaves of *Mangifera indica, *leaves of *Carica papaya, *leaves of *Cassia occidentalis, *and leaves of *Ziziphus mauritiana *	Malaria		+	
		Fruit	Eat three fruits	Constipation	+		

*Manihot esculenta* Crantz (Euphorbiaceae) UNISGSEN07	Manioc (f) Gnambi (w) Mañok (m)	Leaf	Drink the infusion together with milk curdle and then massage the chest	Lung cancer		+	1
			Make aerosol with the infusion	Asthma		+	

		Leaf	Dry in the shade, powder, and eat with food	Diabetes	+		12
		Leaf	Dry it in the shade and then crush it and eat in on rice	Diabetes	+		
		Seed	Dry the seeds on the fire, powder, mix with water, and drink	Diabetes	+		
*Moringa oleifera *Lam. (Moringaceae) UNISGSEN39	Moringa (f) Nebedaay (w) Nebedayo (m)	Root	Grind the root and put it on the fire then dab and bandage	Sore knees	+		
		Bark	Soak the root in water for few seconds and then drink	Sore neck		+	
		Leaf	Put the leaf in water and wash your eyes with it	Eye allergies	+		
		Bark	Soak it in warm (not boiling) water and then drink it	Kidney problems		+	
		Root	Attach the root to the affected area, with *Elaeis guineensis* oil. Do not leave it too long or the root will cause an infection on the sore part.	Rheumatism		+	
		Leaf	Dry the leaves in the shade and eat it with *Phaseolus vulgaris *	Blood pressure	+		
		Seed	Put the seeds on the fire, grind into a powder and drink with water	Blood pressure	+		

		Leaf	Cut the leaves and dry them in the sun for 24 hours	General health	+		4
*Musa paradisiaca * L. (Musaceae)	Bananier (f)	Fruit	Eat it	Stomachache	+		
		Leaf	Infusion with *Combretum micranthum* and *Annona senegalensis *	Blood pressure		+	
			Add to water: dry leaves of *Musa paradisiaca, *little unripe fruits and leaves of* Citrus limon, *leaves of *Mangifera indica, *leaves of *Carica papaya, *leaves of *Cassia occidentalis,* and leaves of *Ziziphus mauritiana *	Malaria		+	

		Leaf	Drink the infusion	Headache		+	3
*Ocimum basilicum * L. (Lamiaceae) UNISGSEN13	Basilic (f) Ngungun (m)	Seeds	Put the seed into the eyes and everything comes out	Eye problems		+	
		Leaf?	Use it with *Calotropis procera *	Rheumatism	+		

*Panicum miliaceum *L. (Poaceae)	Mil (f)	Fruit	Use the powder (pollen) that falls down during the harvest to massage the body with water and salt. Leave it on half an hour and then wash it away.	Allergies		+	2
			Apply millet powder together with lemon juice (*Citrus limon*) on the affected area	Herpes	+		

			Cut the bark into pieces, boil, and use the water as mouthwash	Toothache	+		2
*Parinari macrophylla *Sabine (Chrysobalanaceae)	New (w) Tamba	Bark	Make an infusion and drink	Sore throat		+	
			Make an infusion and drink it before meals	Helps in digestion		+	

			Put the leaf powder on the burns	Burns	+		5
		Leaf	Eat the leaves together with *Carica papaya *fruit	Yellow fever	+		
*Parkia biglobosa* (Jacq.) R.Br. ex G. Don (Fabaceae) UNISGSEN87	Minosa purpre (f) Uul (w) Nete, Nere (m)		Drink the infusion with powdered leaf and milk	Ulcer	+		
			Use it together with *Acacia albida *and *Annona senegalensis *	Depression		+	
			Use it with *Acacia albida, Guiera senegalensis, Annona senegalensis, Adansonia digitata, Soora *(nonidentified plant), and *Ficus sycomorus *	Headache, sore throat, and cold taken as result of the wind		+	
		Seed	Cook them with rice	Diabetes	+		

			Use with *Hibiscus sabdariffa *	Fatigue	+		3
*Phaseolus vulgaris* L. (Fabaceae) UNISGSEN35	Harricot blanche (f) Niebè (w) Ñebbe	Seeds	Make an infusion with seven seeds and eat them	Breast cancer		+	
			Use it with *Hibiscus sabdariffa *	Anaemia	+		
		Leaf?	Eat it together with *Moringa oleifera*'s sundried leaves	Blood pressure	+		

			Put the red liquid inside the bark on the wound then powder the bark and put it on the wound	Wounds	+	+	3
*Piliostigma reticulatum *(DC.) Hochst. (Fabaceae) UNISGSEN77	Fara (m) Kankuran (p)	Bark	Use it with *Annona senegalensis *	Tuberculosis		+	
			Drink the infusion	Chronic cough		+	

*Piper nigrum *L. (Piperaceae)	Poivre noir (f) Mex Pobare (w)	Seed	Drink boiled milk with pepper in it	Sore throat	+		

*Prosopis africana *(Guill. & Perr.) Taub. (Fabaceae)	Yiir (w)	Bark	Drink the decoction	Anaemia	+		1

		Leaf	Make aerosol useful to swat and then drink the water	Vertigo	+		1
*Prosopis juliflora *(Sw.) DC. (Fabaceae) UNISGSEN26	Banaana golo (w)	Leaf	Make aerosol useful to swat and then drink the water	Pregnant women	+		
		Leaf	Drink the infusion	Bellyache		+	

		Young leaf	Make an infusion with *Hibiscus sabdariffa* leaves, drink it, and eat the leaves	Diarrhoea	+	+	10
		Leaf	Drink the infusion	Bellyache		+	
*Psidium guajava * L. (Myrtaceae) UNISGSEN31	Goyave (f) Guyaab (w)		Use it together with *Carica papaya *leaf	Cystitis		+	
		Fruit	Eat the fruit	Bellyache		+	
			Drink the infusion	Diarrhoea		+	
		Leaf	Drink the infusion made with young leaves	Diarrhoea	+		
			Soak the leaves together with *Lawsonia inermis,* and then drink it. The leaves need to be fresh, not dried	Stomachache		+	

*Saba senegalensis* (A.DC.) Pichon (Apocynaceae) UNISGSEN76	Màdd (w) Mat mat (m)	Leaf	Drink the infusion	Intestinal worms	+		2
		Fruit	Boil the fruit	Malnutrition		+	

		Leaf	Make an infusion	Skin fungus	+		5
*Solanum lycopersicum *L. (Solanaceae)	Tomate (f) Tamaate (w)		Crush the leaf and put the juice and the leaf inside the ear	Earache	+		
		Fruit	Apply tomato sauce on the wound	Wounds	+	+	
			Eat it raw	Smallpox	+		

*Solanum tuberosum * L. (Solanaceae)	Pomme de terre (f) Pombiteer (w)	Leaf	Make an infusion and give it to the 1-week-old baby to drink	Strong child	+		1
		Fruit	Eat it raw	Menstrual pain	+		

			Use it with *Combretum micranthum *	Asthma	+		
		Fruit	Boil it and wash your eyes with it	Vision problems		+	4
*Tamarindus indica* L. (Fabaceae)	Tamarin (f) Daqaar (w)		Add salt and rinse your mouth with it	Toothache		+	
			Karitè butter together with the bark of *Tamarindus indica *	Bruises	+		
		Bark	Use it with *Hibiscus sabdariffa* and the bark of *Acacia nilotica *	Fatigue	+		
			Use it with *Combretum micranthum *	Blood pressure	+		

*Tinospora bakis* (A. Rich.) Miers (Menispermaceae)	Bakis (w)	Root	Eat the root in a soup with a ripe *Carica papaya *and chicken	Yellow fever	+		1

			Massage the neck	Sore neck (2)	+	+	11
			Massage the body	Cold	+		
			Put it on the sore part	Bruises	+		
			Massage the chest with Karitè butter	Chronic cough	+		
			Add salt to the butter and dab on the back	Backache	+		
			Karitè butter together with the bark of *Tamarindus indica *	Bruises	+		
			Massage and dab the sore part of the body	Rheumatism	+		
*Vitellaria paradoxa * C.F. Gaertn. (Sapotaceae)	Karitè (f)	Seed→butter	Apply on herpes	Herpes	+		
			Use it with *Carapa procera* and *Elaeis guineensis*' oil	Hair loss	+		
			Use it together with *Carapa procera*'s nut oil and dab it on the body three times a day	Backache		+	
			Dab on the hair	Stronger hair	+		

			Use it together with a stick to apply this part	Fractures	+		
			Add coffee and tyr (red oil)	To get rid of the dead blood, against fatigue	+		
			Use it with *Aloe vera *	Tuberculosis		+	
			Use it with *Aloe vera *	Hair loss	+		

*Xylopia aethiopica* (Dunal) A. Rich. (Annonaceae) UNISGSEN70	Diar (w) Jar	Fruit Seed	The seeds are used to prepare the “Touba Coffee”	Blood pressure	+		3

(f): French; (m): Mandingo; (p): Pulaar; (w): Wolof; Qs: quotations (number of informants, who have quoted a specific taxon); ?: uncertain information.
